# Optical imaging (HandScan) can identify ultrasound remission in rheumatoid arthritis

**DOI:** 10.1186/s12891-024-07472-4

**Published:** 2024-05-07

**Authors:** Charline Rinkin, Olivier Malaise, Florane Chauveheid, Caroline Gerard, Laurence Seidel, Michel Malaise, Clio Ribbens

**Affiliations:** 1 Rheumatology department, University Hospital of Liège, Liège, Belgium; 2 Biostatistics and research method center (B-STAT), University Hospital of Liège, Liège, Belgium

**Keywords:** Optical spectral transmission, Rheumatoid arthritis, Remission, Ultrasonography

## Abstract

**Background:**

Identifying remission is of high importance in rheumatoid arthritis (RA) because remission is associated with less structural progression. We investigated the efficacy of a new optical imaging device, HandScan, to identify RA remission, as defined by ultrasound (US).

**Methods:**

61 RA patients were included. Disease activity was evaluated by clinical assessment and US, using gray-scale (GS) and Power Doppler (PD). HandScan determined unitary optical spectral transmission (OST) values for wrists, metacarpophalangeal and proximal interphalangeal joints. At the patient level, three composite HandScan (HS) scores were calculated: total HS score; disease activity score OST (DAS-OST) and DAS-OST without patient global assessment (PtGA). Using ROC curves, we determined HS cut-offs to identify US-defined remission.

**Results:**

At the joint level, unitary OST values significantly correlated with GS synovitis [odds ratio (OR) 2.43, *p* < 0.0001] and PD positivity (OR 3.72, *p* = 0.0002 ). At the patient level, total HS score and DAS-OST were significantly associated with all gray-scale US (GSUS) and power doppler US (PDUS) parameters evaluated (synovitis number and grade, synovial thickness, PD grade) (*p* < 0.05). The cut-off to identify US-defined remission at the joint level was of 0.92, giving an 81% sensitivity and a 96% positive predictive value (PPV). At the patient level, ROC-curves failed to identify a robust cut-off for the total HS score, but did identify a cut-off (3.68) for DAS-OST to identify US-defined remission, but with lower sensitivity (75%), specificity (56%) and PPV (67%).

**Conclusions:**

HandScan is a non-invasive optical imaging technique providing OST values that correlate with GSUS and PDUS parameters. In addition, HandScan is able to reliably identify US-defined remission in RA at the joint level, with a good sensitivity and high PPV. At the patient level, HandScan DAS-OST can also determine US remission (while total HS score failed to do so), but with lower performance.

**Supplementary Information:**

The online version contains supplementary material available at 10.1186/s12891-024-07472-4.

## Background

Rheumatoid arthritis (RA) is a chronic inflammatory disease in which the primary lesion is the inflamed synovial membrane, characterized by hyperplasia, infiltration by immune cells, neo-angiogenesis and fibrosis [1]. Accurate assessment of joint inflammation is important because its presence is associated with structural damage and poor functional prognosis. Ultrasonography (US) is a reliable tool to evaluate joint inflammation and is readily available in clinical practice. US is more sensitive than clinical evaluation to detect synovitis [2] and power Doppler (PD) positivity is a risk factor for flare even if patients are considered to be in clinical remission [3]. However, US evaluation is time-consuming and can only be performed by trained physicians.

**Fig. 1:**
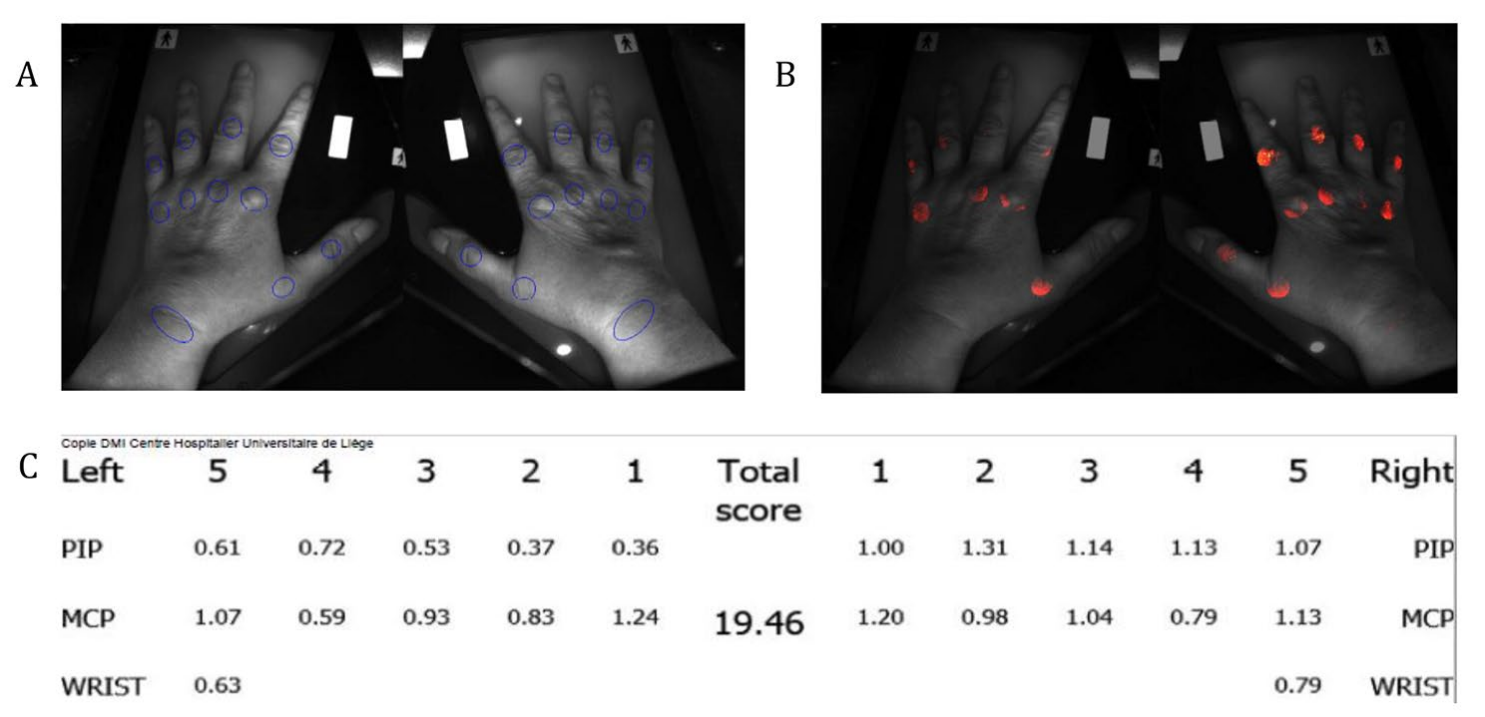
HandScan (HS) procedure. (**A**) Regions of interest corresponding to the 22 studied joints. (**B**) Colorimetric results representing the light attenuation for each joint. (**C**) Optical spectral transmission (OST) value for each joint and the total HS score. PIP = proximal interphalangeal joint; MCP = metacarpophalangeal joint

HandScan (HS) is a new imaging device using optical spectral transmission (OST), allowing non-invasive assessment of inflammatory activity in wrists and hands. It is based on the specific absorption by blood of the light transmitted within a tissue. In joints with synovitis, the light transmission is decreased. For each joint, a computer algorithm translates the light absorption into a value from 0 to 3 and into an image that can be easily interpreted by the practitioner, with a color scale starting from black, with an increasing level of inflammation ranging from red (very low) to yellow (moderate) then white (high) (Fig. 1). Advantages of HandScan are the reproducibility, with good inter- and intra-observer values, and the lack of pain [4, 5], that are also characteristics of US. Furthermore, the acquisition of HandScan (1.5 min) is faster than an US examination. In contrast to US, HandScan can be performed by a medical assistant and does not require the presence of a physician, while US requires an experimented and well-trained physician. US reliability follows a learning curve and also depends on machine performances.

Previous studies have shown that, at the joint level, OST values correlate (even if weakly) with the clinical evaluation [6]. At the patient level, the HandScan activity is represented by a total OST score (“total HS score”) [the score ranging from 0 to 66 for the 22 joints evaluated: wrists, metacarpophalangeal (MCP) joints and proximal interphalangeal (PIP) joints]. This total optic score was significantly higher in patients with high disease activity than in those in remission or low/moderate disease activity [7]. A longitudinal association between the total HS score and the disease activity score (DAS) of 28 joints (DAS28) has been found, but the explained variance is quite low [6]. In 2022, Verhoeven et al. developed composite index scores based on OST: DAS-OST, and DAS-OST without patient global assessment (PtGA) on a visual analogue scale (VAS) (PtGA-VAS) [8]. They concluded that the DAS-OST score seems to be the most accurate to monitor the patient’s disease activity, with good negative and positive predictive values for clinical remission. HandScan has also been described as effective to assess response to glucocorticoid therapy in patients with arthritis [9] and able to classify RA patients into active or inactive [10].

While several studies have compared HandScan to clinical evaluation, only a few have compared it to US. It has been shown that, at the joint level, OST value correlates with joint inflammation [4, 5, 11, 12]. At the patient level, total HS score moderately correlates with the gray-scale US (GSUS) and power Doppler US (PDUS) [5, 11, 13]. However, no OST threshold has been determined to identify US-defined remission at the patient level.

The objective of this study is to analyze the HandScan performance to evaluate US-defined remission in RA patients. We aimed to analyze correlations between US and HandScan parameters and to determine HandScan cut-off values to predict US-defined remission both at the joint level and at the patient level.

## Methods

### Study design and patients

Sixty-one patients with RA fulfilling the ACR/EULAR 2010 criteria [14] were recruited at the outpatient clinic of the rheumatology department of the University Hospital of Liege between October 2018 and April 2021. The study was approved by the ethics committee of the hospital (B70720108722), and written informed consent was obtained from each patient. The study consisted of assessment of disease activity by clinical examination, composite disease activity scores, HandScan (that can only assess hands and wrists) and US of hands and wrists. All joint assessments were performed on the same day by three independent investigators (one for the clinical examination, one for the US and one for the HandScan), blinded for other outcomes. Exclusion criteria were similar to those of other studies evaluating HandScan [4, 5, 11, 12]: major hand deformity, recent surgery of the hands or presence of prosthetic material in the hands. Children and patients with cutaneous psoriasis were also excluded.

Subjective assessments included the patient (PtGA) and the physician (PGA) global assessments on a visual analogue scale (VAS) (0–100 mm) and the Health Assessment Questionnaire (HAQ) [15]. Clinical examination included the number of tender and swollen joints. Blood samples were obtained for evaluation of rheumatoid factor, antibodies to citrullinated proteins (ACPAs), C-reactive protein (CRP) and erythrocyte sedimentation rate (ESR). Disease activity was evaluated using DAS28-CRP and DAS28-ESR [16], Simplified Disease Activity Index (SDAI) [17] and Clinical Disease Activity Index (CDAI) [18]. Remission cut-off levels were those used in literature (DAS28-CRP and DAS28-ESR remission if ≤ 2.6; CDAI remission if ≤ 2.8; SDAI remission if ≤ 3.3; Boolean remission if tender joint ≤ 1 and swollen joint ≤ 1 and CRP ≤ 1 mg/dL and PtGA ≤ 1/10 [19]).

### Ultrasonography

Each US was performed by one experienced examiner using a 10–14 MHz B-mode multifrequency transducer (Logiq E9, GE Healthcare, Milwaukee, WI, USA). GSUS and PDUS were carried out on 22 joints for each patient (wrists: radiocarpal and intercarpal; MCP joints 1–5 and PIP joints 1–5). Patient and probe positioning were used as recommended by EULAR guidelines [20]. Synovitis was classified according to OMERACT [21] (definition, measurement of grade from 0 to 3 in GS and in PD). In joints where 2 scans were made (wrists), the joint was considered positive if at least one measurement was positive. The following parameters were collected at the joint level, as in our previous work [22]: presence of synovitis, synovitis grade, synovial thickness (mm), presence of PD and PD grade. At the patient level, the following US parameters were collected: number of joints with synovitis, sum of grade of the 22 joints (cumulative synovitis grade in gray score), mean synovitis grade, cumulative synovitis thickness (the sum of thickness of all US-positive joints, mm), mean synovitis thickness (mm), number of PD-positive joints and cumulative PD grade. We also looked for the presence of tenosynovitis of the wrists and hands [23]. Their presence or absence was documented in a binary way. We defined US remission as did Besselink et al. [4]: remission was defined, at the joint level, as “GSUS synovitis ≤ 1 and PDUS synovitis 0”; remission was defined, at the patient level, as “GSUS synovitis ≤ 1 and PDUS synovitis 0 and GSUS/PDUS tenosynovitis 0”.

### Optical transmission measurements

HandScan [Hemics (Eindhoven, the Netherlands)] was carried out by a rheumatology nurse. Forearms were inserted into cylindrical openings containing armbands. Hands laid flat on a glass surface. Lights with wavelengths of 660 and 808 nm illuminated the same 22 joints as those analyzed by US (PIPs, MCPs and wrists). The light transmitted through the joints and the reference regions were continuously recorded on the dorsal side by a camera. A complete measurement was performed in ± 100 s. Cuffs were first inflated to 5 mmHg (± 10 s), then to 55 mmHg (± 60 s) and were finally deflated (± 30 s). The size and position of the regions of interest were defined automatically by the computer software of the device.

The HandScan (HS) software automatically calculates a unit joint value (i.e. OST value) which ranges from 0 to 3 for each of the 22 joints studied (0 corresponding to a total absence of inflammatory activity and 3 to maximum inflammatory activity) and an overall total score (i.e. total HS score), which corresponds to the average score per joint multiplied by 22, ranging from 0 to 66. At the patient level, we also calculated DAS-OST score and DAS-OST without PtGA score according to Verhoeven et al. [8] (DAS OST : -0.44 + OST*0.03 + male*-0.11 + LN (ESR) * 0.77 + PtGA * 0.03 ; DAS-OST without PtGA : -0.11 + OST*0.04 + male*-0.25 + LN (ESR) * 0.88). For total HS score, the clinical remission threshold proposed by Besselink et al. is a total HS score ≤ 11 with no more than one joint with unitary OST score > 1 [24]. For the DAS-OST and DAS-OST without PtGA, the clinical remission threshold was ≤ 2.6 [8].

### Statistical analysis

Results are presented as mean ± standard deviation (SD) for continuous variables and as frequency tables for qualitative variables. At the joint level, logistic regression or ordinal regression [odds ratio (OR) and 95% confidence interval (CI) OR] was used to study the presence or the grade according to a parameter, while regression linear (r) was used to study the relationship between two continuous variables. To study the relationship between the HandScan parameters at the patient level (total HandScan score, DAS OST and DAS OST without PtGA) and the other parameters (clinical or ultrasound), we used the logistic regression model if the test was binary, the ordinal logistic regression model if the test was an ordinal variable, the linear regression model if the test was a quantitative variable, the Poisson regression model for counts and the Tweedie regression model for continuous variables including many zero values. These models were adjusted for parameters that could influence optical spectral transmission, namely age, sex, smoking, BMI, Raynaud’s phenomenon and use of β-blockers. For each model, we report the β coefficient and its standard error as well as the *p*-value. Sensitivity, specificity, accuracy, positive predictive value (PPV), negative predictive value (NPV) and their 95% CI were used to compare a threshold (defined in the literature or the one we calculated), with respect to a gold-standard characterizing disease remission. To determine a new threshold, ROC (Receiver Operating Characteristic) curves were calculated. The results are considered significant at the 5% level of uncertainty (*p* < 0.05). Calculations were performed using SAS version 9.4.

## Results

### Patient characteristics and HandScan results

61 RA patients were studied (44 women, 17 men). Table 1 describes patient demographics, characteristics of rheumatoid disease, characteristics that could influence optical transmission (BMI, active smoking, Raynaud’s phenomenon, treatment with beta-blockers), evaluation of RA activity as well as US characteristics. Patients on glucocorticoids were taking low doses of daily oral prednisolone (mean dose of 2 ± 3 mg). At the patient level, HandScan results are provided by the software as the total HS score: mean total HS score was 12.8 ± 6.3 (*n* = 61). We also calculated two OST scores using ESR validated from literature [8]: mean DAS-OST was 3.2 ± 1.0 (*n* = 59) and mean DAS-OST without PtGA 2.5 ± 0.8 (*n* = 59).

**Table 1:** Clinical and ultrasound characteristics of the RA patient population. ACPA: anti-citrullinated peptide antibody; DMARD: disease modifying anti-rheumatic drug; NSAID: non-steroidal anti-inflammatory drug; number; SD: standard deviation; VAS: visual analogue scale; CRP: C-reactive protein; ESR: erythrocyte sedimentation rate; DAS: disease activity score; CDAI: clinical disease activity index; SDAI: simple disease activity index; PD: power doppler

Variable (per patient)	*N*	Mean ± SD, Median (min-max) or Number (%)	Variable (per patient)	*N*	Mean ± SD, Median (min-max) or Number (%)
Patients’ characteristics					
Female gender	61	44 (72)	Glucocorticoids	61	26 (43)
Age (year)	61	62 (27–81)	NSAIDs	61	13 (21)
Disease duration (year)	61	7 (0–30)	Body mass index (kg/m^2^)	61	26 (17–41)
Rheumatoid factor	61	41 (67)	Active smoking	61	18 (29)
ACPA	61	42 (69)	Raynaud’s phenomenon	61	6 (10)
Conventional DMARDs	61	40 (66)	Beta-blockers	61	15 (25)
Biological DMARDs	61	32 (52)			
Subjective evaluation of disease activity			Ultrasound characteristics		
Patient global assessment - VAS (mm)	61	48.61 ± 27.83	Number of joints with synovitis	61	3.10 ± 4.55
Physician global assessment - VAS (mm)	61	23.12 ± 24.68	Cumulative synovitis grade	61	5.38 ± 9.42
Health Assessment Questionnaire (/60)	61	16.59 ± 11.84	Mean synovitis grade	61	0.24 ± 0.43
Blood inflammatory parameters			Cumulative synovial thickness (mm)	61	7.01 ± 11.53
CRP (mg/L)	61	7.33 ± 15.90	Mean synovial thickness (mm)	61	0.32 ± 0.52
ESR (mm/h)	59	17.24 ± 16.03	Number of PD-positive joints	61	0.25 ± 0.70
Clinical examination			Cumulative PD grade	61	0.43 ± 1.50
Number of swollen joints / patient	61	3.16 ± 4.43	Mean PD grade	61	0.02 ± 0.07
Number of tender joints / patient	61	5.67 ± 6.29	Tenosynovitis	61	
Disease activity index			No		48 (79)
DAS28-CRP	61	3.61 ± 1.38	Yes		13 (21)
Remission		14 (23)	Absence of US remission	61	
Low activity		12 (20)	No		32 (52)
Moderate activity		27 (44)	Yes		29 (48)
High activity		8 (13)			
DAS28-ESR	59	3.82 ± 1.36			
Remission		12 (20)			
Low activity		9 (15)			
Moderate activity		27 (46)			
High activity		11 (19)			
CDAI	61	16.01 ± 13.74			
Remission		9 (15)			
Low activity		18 (29)			
Moderate activity		16 (26)			
High activity		18 (29)			
SDAI	61	16.74 ± 13.99			
Remission		10 (16)			
Low activity		19 (31)			
Moderate activity		19 (31)			
High activity		13 (21)			
Boolean remission	61				
No		51 (84)			
Yes		10 (16)			

### Correlation between OST and US

First, we analyzed associations between OST values and US parameters at the joint level (Table 2). Unitary OST values significantly correlated with GSUS synovitis (presence of synovitis, synovitis grade and synovial thickness) and PDUS (PD presence and grade). Analysis per type of joint found these correlations to be present for MCPs and PIPs, but not for wrists (with the exception of grade of synovitis). Table 3 shows the beta coefficients found by regression models at patient level between OST scores (total HS score, DAS-OST, DAS-OST without PtGA) and US parameters in a multivariate analysis, after adjustment for demographic data and factors that could influence optical transmission (age, gender, smoking, BMI, Raynaud’s phenomenon and treatment with beta-blockers). Both total HS score and DAS-OST were significantly associated with GSUS synovitis (number and grade), synovial thickness, PDUS (number of PD-positive joints and cumulative PD grade), as well as with absence of US remission at the patient level. Total HS score also correlated with tenosynovitis.

**Table 2:** Association, at the joint level, between the unitary OST value and US parameters Odds ratio (OR) were calculated for synovitis, synovitis grade, PD positivity, PD grade, while correlation coefficient (r) was calculated for synovial thickness (continuous variable). MCP: metacarpophalangeal joint; PIP: proximal interphalangeal joint; PD: power doppler

	All joints (*n* = 1320)		Wrists (*n* = 120)		MCP (*n* = 600)		PIP (*n* = 600)	
	OR / r	*P*-value	OR / r	*P*-value	OR / r	*P*-value	OR / r	*P*-value
Synovitis	**2.43**	**< 0.0001**	*1.69*	*0.22*	**2.52**	**0.0001**	**2.05**	**0.044**
Synovitis (grade)	**3.80**	**< 0.0001**	**2.55**	**0.047**	**3.82**	**< 0.0001**	**5.33**	**0.0053**
Synovial thickness	**0.26**	**< 0.0001**	*0.27*	*0.061*	**0.36**	**< 0.0001**	**0.23**	**0.002**
PD positivity	**3.72**	**0.0002**	*3 0.38*	*0.21*	*2.46*	*0.25*	**44.80**	**0.0009**
PD grade	**3.71**	**< 0.0001**	*3.36*	*0.20*	**2.90**	**0.0083**	**42.40**	**0.0013**

**Table 3:** Association (regression models with beta coefficients, standard error and *p*-values), at the patient level, between OST scores (Total HS score, DAS-OST and DAS-OST without PtGA) and US parameters. HS: HandScan; DAS: disease activity score; OST: optical spectral transmission; PtGA = patient global assessment; SE: standard error; PD: power doppler; US: ultrasounds

	Total HS score		DAS-OST		DAS-OST without PtGA	
Variable	Beta coefficient (SE)	*P*-value	Beta coefficient (SE)	*P*-value	Beta coefficient (SE)	*P*-value
Number of synovitis	**0.13 (0.013)**	**< 0.0001**	**0.42 (0.092)**	**< 0.0001**	*0.14 (0.11)*	*0.19*
Cumulative synovitis grade	**0.15 (0.026)**	**< 0.0001**	**0.48 (0.18)**	**0.0064**	*0.26 (0.24)*	*0.28*
Mean synovitis grade	**0.16 (0.028**	**< 0.0001**	**0.53 (0.19)**	**0.0047**	*0.19 (0.25)*	*0.46*
Cumulative synovial thickness (mm)	**0.16 (0.028**)	**< 0.0001**	**0.53 (0.19)**	**0.0047**	*0.19 (0.25)*	*0.46*
Mean synovial thickness (mm)	**0.15 (0.026)**	**0.0001**	**0.48 (0.18)**	**0.0064**	*0.26 (0.24)*	*0.28*
Number of PD positive joint	**0.18 (0.073)**	**0.016**	**0.99 (0.39)**	**0.0113**	*0.66 (0.45)*	*0.15*
Cumulative PD grade	**0.22 (0.082)**	**0.0073**	**1.10 (0.48)**	**0.022**	*0.74 (0.59)*	*0.21*
Mean PD grade	**0.22 (0.085)**	**0.0096**	**1.10 (0.48)**	**0.021**	*0.74 (0.58)*	*0.20*
Tenosynovitis	**0.24 (0.087)**	**0.0056**	*-0.12 (0.38)*	*0.74*	*0.053 (0.49)*	*0.91*
Absence of US remission	**0.21 (0.078)**	**0.0065**	**0.79 (0.34)**	**0.022**	**1.00 (0.42)**	**0.018**

### Diagnostic performance of OST to identify US-defined remission

At the joint level, US remission was defined as GSUS synovitis ≤ 1 and PDUS synovitis 0. First, we used ROC-curves to identify our local optimal cut-off for OST value to determine remission for any individual joint or for each joint subtype (PIP, MCP and wrist). Sensitivity, specificity, accuracy, PPV and NPV were calculated. The OST cut-off for assessing US remission was 0.92 when considering all 22 joints together, 0.95 for wrist, 0.70 for MCP and 0.99 for PIP. Table 4 displays the diagnostic performance of HandScan to detect US remission at the joint level. Our OST cut-off had a high sensitivity and a high PPV to identify joint remission (sensitivity: 80.5%, CI: 78.2–82.6; PPV 95.9%, CI: 94.5–97.0), meaning that our OST cut-off was able to predict that an individual joint was under US remission. When evaluating each joint separately, PPV was also high for all the joint subtypes (96.3, 97.5 and 91.1% for PIP, MCP and wrist respectively). Accuracy was significantly better for PIP than for MCP or wrist (87.2%, versus 64.5% and 75.4%).

**Table 4:** Diagnostic performance of HandScan to identify US remission at the joint and at the patient levels. US-remission, at the joint level, was defined as GSUS synovitis ≤ 1 and PDUS synovitis = 0. US remission, at the patient level, was defined as GSUS synovitis ≤ 1 and PDUS synovitis 0 and GSUS/PDUS tenosynovitis 0. CI: confidence interval; OST: optical spectral transmission; MCP: metacarpophalangeal joint; PIP: proximal interphalangeal joint; HS = HandScan; DAS = disease activity score

	Sensitivity % (95% CI)	Specificity % (95% CI)	Accuracy % (95% CI)	Positive predictive value % (95% CI)	Negative predictive value % (95% CI)
At the joint level					
**“Any joint” OST score (cut-off 0.92)**	**80.5** **(78.2–82.6)**	**57.4** **(47.2–67.2)**	**78.7** **(76.4–80.9)**	**95.9** **(94.5–97.0)**	**19.3** **(15.0-24.3)**
Wrist OST score (cut-off 0.95)	78.9 (69.7–86.2)	55.6 (30.8–78.5)	75.4 (66.8–82.8)	91.1 (83.2–96.1)	31.3 (16.1–50.0)
MCP OST score (cut-off 0.70)	63.0 (58.9–67.1)	81.6 (68.0-91.2)	64.5 (60.6–68.3)	97.5 (95.3–98.9)	16.2 (11.8–21.4)
PIP OST score (cut-off 0.99)	89.9 (87.2–92.3)	41.2 (24.7–59.3)	87.2 (84.3–89.7)	96.3 (94.3–97.7)	19.4 (11.1–30.5)
At the patient level,					
Total HS score	/				
DAS-OST (cut-off 3.68)	75.0 (56.6–88.5)	55.6 (35.3–74.5)	66.1 (52.6–77.9)	66.7 (49.0-81.4)	65.2 (42.7–83.6)

At the patient level, global US remission was defined as “GSUS synovitis ≤ 1 and PDUS synovitis 0 and GSUS/PDUS tenosynovitis 0”. We determined with ROC-curves a cut-off to estimate US remission with HandScan (Table 4). For the total HS score, ROC curves failed to identify a robust cut-off. For DAS-OST score, we identified a DAS-OST cut-off of 3.68. Diagnostic performance of HandScan to identify US remission at the patient level was lower, with at the best a 75.0% (56.6–88.5) sensitivity (Table 4).

### Diagnostic performance of OST to identify clinical remission at the patient level

We also analyzed HandScan performance to identify clinical remission in our RA cohort (clinical remission was defined according to DAS28-CRP, DAS28 ESR, SDAI, CDAI and Boolean remission). We used cut-off from literature for total HS score (remission if total HS score ≤ 11 with no more than one joint with unitary OST score > 1) and DAS-OST score (remission if ≤ 2.6) to analyze if HandScan can identify clinical remission (“literature cut-off”). We also established ROC-curves to identify our optimal local optimal cut-off for these two OST scores (“local cut-off”). We demonstrated high NPV for clinical remission detection with both total HS score and DAS-OST scores, meaning that these two scores are highly able to identify patients that are not under clinical remission: e.g. for DAS28-CRP remission, NPV were 90.7% (CI: 77.9–97.4) and 94.9% (CI: 82.7–99.4) for the cut-off from literature or the local cut-off respectively (Supplementary Table 1). As observed for US parameters, DAS-OST was more accurate than the total HS score if we consider the clinical remissions scores (Supplementary Table 1): e.g. for DAS28-CRP remission as a reference, accuracy for estimating DAS-OST remission was 83.1% (CI: 71.0-91.6) with the cut-off from literature as well as with the local cut-off, while accuracy for estimating total HS score remission was only 70.5 (CI: 57.4–81.5) and 68.9% (CI: 55.7–80.1) with the cut-off from literature or the local cut-off respectively.

Lastly, we analyzed associations between OST scores and disease activity (at the patient level) in multivariate analysis. Both total HS score and DAS-OST significantly correlated with clinical evaluation and disease activity score (Supplementary Table 2): DAS-OST and total HS score were significantly associated with number of swollen or tender joints, HAQ, physician VAS, DAS28-CRP, DAS28-CRP remission, Boolean remission, CDAI, SDAI and SDAI remission. DAS-OST was also associated with patient VAS, DAS28-ESR and DAS28-ESR remission. In contrast, DAS-OST without PtGA was only associated with DAS-ESR remission.

## Discussion

Our study demonstrates that HandScan can be used to identify US-defined remission in RA both at the joint level and at the patient level, and that HandScan cut-off values can be used to determine remission in an individual RA patient.

At the joint level, unitary OST values correlate with GSUS and PDUS parameters. Correlations were present for MCPs and PIPs, but not for the wrists. At the patient level, we found similar observations, i.e. correlations between the total HS score on one hand and GSUS/PDUS on the other hand. In addition, correlations were present at the patient level between global US activity score and total HS score. Our results are in accordance with those of Besselink et al., van Onna et al., Triantafyllias et al. and Blanken et al. [4, 5, 11, 13], who found correlations between US and OST scores. Besselink et al. underlined that these correlations were better at the joint level than at the patient level [4]. In addition to the total HS score, we analyzed the DAS-OST score (that is estimated to be more accurate to monitor patient’s disease according to Verhoeven et al. [8]) and found significant correlations between DAS-OST score and synovitis, PD and US global activity, while this was not the case for DAS-OST without PtGA.

We determined a cut-off for the HandScan to identify US-defined remission at the joint level. We established a cut-off for each joint type (wrist, MCP and PIP), as well as for the 22 joints evaluated together. These cut-offs showed a high positive predictive value, meaning that if a unitary joint is under remission according to the HandScan remission, we can reliably ascertain that this joint will also be in remission according to US evaluation. Of interest, we established a unique cut-off that can be used for the 22 joints evaluated together, also with a high PPV. The good sensitivity and high positive predictive value at the joint level can position HandScan as a screening tool in clinical practice: if all joints are under the remission HandScan cut-off, the patient does not need a further outpatient evaluation by a rheumatologist Previous work by Krabbe et al. also investigated cut-offs to determine US unitary joint activity [12]. In their analysis, sensitivity was better for the wrist US remission, while specificity was better for MCPs, PIPs and all joints together.

We further determined a HandScan cut-off to identify US-defined remission at the patient level. Our cut-off for DAS-OST has a 75% sensitivity to identify US-defined remission, with a definition that includes synovitis and tenosynovitis (remission if GSUS ≤ 1, no PD, no tenosynovitis). No cut-off was found in our cohort for the total HS score, indicating that DAS-OST is better than total HS score to assess US remission.

In addition to US-defined remission, we also analyzed the performance of HandScan to assess clinical-defined remission. We confirm that HandScan can be used to identify RA patients under clinical remission, with a good efficacy and a high NPV in our cohort. NPV was high, while PPV was far lower: this was also observed by Verhoeven et al. in their first cohort for Boolean remission and in their replicative cohort for DAS28 remission and low disease activity [8]. For clinical remission, DAS-OST without PtGA did not discriminate between patients under remission or not (according to DAS28-CRP, CDAI, SDAI and Boolean remission). This is in accordance with Verhoeven et al. who demonstrated lower performances for DAS-OST without PtGA than with DAS-OST [8]. We also found DAS-OST to be more efficient that the total HS score to determine if patients were under remission or not: accuracy was better for DAS-OST than for total HS score for each remission definition, and while a cut-off was determined in our cohort for each remission definition with DAS-OST, this was not the case for the total HS score and the remission definitions using DAS28-ESR and CDAI.

A limitation of our study is the lack of X-rays, since the presence of osteoarthritis has been described to influence the optical transmission [4]. The other factors that could influence light transmission (such as age, sex, BMI, Raynaud phenomenon, beta-blockers or smoking) were taken in account in the multivariate analysis. Another limitation of this study is that HandScan is per se limited to hands and wrists and that some patients considered under remission by HandScan can still have active joints elsewhere, e.g. in the feet.

## Conclusions

HandScan is a non-invasive optical imaging technique, significantly associated with GSUS and PDUS parameters, both at the joint and at the patient level. With regard to the ability of HandScan to identify US-defined remission, at the joint level, OST values can determine US remission with a good specificity and high positive predictive value. HandScan could therefore be used as a tool in clinical practice as a first-hand evaluation by a healthcare worker, and identify patients not needing further evaluation by the rheumatologist if all joints are under the remission HandScan cut-off, thereby saving patients’ and rheumatologists’ time.

### Electronic supplementary material

Below is the link to the electronic supplementary material.

Supplementary Material 1

## Data Availability

The datasets used and/or analyzed during the current study are available from the corresponding author on reasonable request.

## Abbreviations

ACPAs: antibodies to citrullinated proteins
BMI: body mass index
CDAI: clinical disease activity Index
CI: confident interval
CRP: C-reactive protein
DAS: disease activity score
DMARDs: disease modifying antirheumatic drugs
ESR: erythrocyte sedimentation rate
GSUS: gray-scale ultrasonography
HS: HandScan
JC: joint count
MCP: metacarpophalangeal
NPV: negative predictive value
OR: odds ratio
OST: optical spectral transmission
PDI: power doppler index
PDUS: power Doppler ultrasonography
PGA: physician global assessments
PtGA: patient global assessment
PPV: positive predictive value
RA: rheumatoid arthritis
ROC: receiver operating characteristic
SDAI: simple disease activity index
US: ultrasonography
VAS: visual analogue scale
