# Supplementary material for: Optical imaging (HandScan) can identify ultrasound remission in rheumatoid arthritis
Source: BMC Musculoskelet Disord. 2024 May 7;25:361. doi: 10.1186/s12891-024-07472-4 (PMC11075232; doi:10.1186/s12891-024-07472-4)
Supplement: Supplementary file 1 — Supplementary Material 1 [file 12891_2024_7472_MOESM1_ESM.docx]

**SUPPLEMENTARY INFORMATION**

**Additional file 1: Supplementary Table 1. Diagnostic performance of total HS score and DAS-OST to identify remission (local cut-off adapted for each score and cut-off from literature).** CI = confidence interval; HS= HandScan; DAS= disease activity score; OST= optical spectral transmission. In light grey, value > 80%; in dark grey, value > 90%. Diagnostic performance of total HS score cannot be evaluated for DAS28-ESR and CDAI because lack of correlation.

**Additional file 2: Supplementary Table 2. Association (regression models with beta coefficients, standard error and p-values) at the patient level, between OST results (Total HS score, DAS-OST and DAS-OST without PtGA) and disease characteristics (multivariate analysis).**

**Supplementary Table 1. Diagnostic performance of total HS score and DAS-OST to identify remission (local cut-off adapted for each score and cut-off from literature).** CI = confidence interval; HS= HandScan; DAS= disease activity score; OST= optical spectral transmission. In light grey, value > 80% ; in dark grey, value > 90%. Diagnostic performance of total HS score cannot be evaluated for DAS28-ESR and CDAI because lack of correlation.

|  | Sensitivity  % (95% CI) | Specificity  % (95% CI) | Accuracy  % (95% CI) | Positive predictive value  % (95% CI) | Negative predictive value  % (95% CI) |
| --- | --- | --- | --- | --- | --- |
| **DAS28-CRP remission as a reference** | | | | | |
| Total HS score (cut-off from literature) | 50.0  (23.0-77.0) | 76.6  (62.0-87.7) | 70.5  (57.4-81.5) | 38.9  (17.3-64.3) | 83.7  (69.3-93.2) |
| Total HS score (local cut-off for DAS28-CRP, <9.26) | 71.4  (41.9-91.6) | 68.1  (52.9-80.9) | 68.9  (55.7-80.1) | 40.0  (21.1-61.3) | 88.9  (73.9-96.9) |
| DAS-OST (cut-off from literature) | 71.4  (41.9-91.6) | 86.7  (73.2-95.0) | 83.1  (71.0-91.6) | 62.5  (35.4-84.8) | 90.7  (77.9-97.4) |
| DAS-OST (local cut-off for DAS28-CRP, <2.73) | 85.7  (57.2-98.2) | 82.2  (68.0-92.0) | 83.1  (71.0-91.6) | 60.0  (36.1-80.9) | 94.9  (82.7-99.4) |
| **DAS28-ESR remission as a reference** | | | | | |
| Total HS score (cut-off from literature) | / | | | | |
| Total HS score (local cut-off for DAS28-ESR) | / | | | | |
| DAS-OST (cut-off from literature) | 66.7  (34.9-90.1) | 83.0  (69.2-92.4) | 79.7  (67.2-89.0) | 50.0  (24.7-75.4) | 90.7  (77.9-97.4) |
| DAS-OST (local cut-off for DAS28-ESR, <2.45) | 66.7  (34.9-90.1) | 97.9  (88.7-99.9) | 91.5  (81.3-97.2) | 88.9  (51.8-99.7) | 92.0  (80.8-97.8) |
| **SDAI remission as a reference** | | | | | |
| Total HS score (cut-off from literature) | 50.0  (18.7-81.3) | 74.5  (60.4-85.7) | 70.5  (57.4-81.5) | 27.8  (9.7-53.5) | 88.4  (74.9-96.1) |
| Total HS score (local cut-off for SDAI, <11.0) | 90.0  (55.5-99.8) | 58.8  (44.2-72.4) | 63.9  (50.6-75.8) | 30.0  (14.7-49.4) | 96.8  (83.3-99.9) |
| DAS-OST (cut-off from literature) | 80.0  (44.4-97.5) | 83.7  (70.3-92.7) | 83.1  (71.0-91.6) | 50.0  (24.7-75.4) | 95.4  (84.2-99.4) |
| DAS-OST (local cut-off for SDAI, <2.73) | 100.0  (69.2-100.0) | 79.6  (65.7-89.8) | 83.1  (71.0-91.6) | 50.0  (27.2-72.8) | 100.0  (91.0-100.0) |
| **CDAI remission as a reference** | | | | | |
| Total HS score (cut-off from literature) | / | | | | |
| Total HS score (local cut-off for CDAI) | / | | | | |
| DAS-OST (cut-off from literature) | 88.9  (51.8-99.7) | 84.0  (70.9-92.8) | 84.8  (73.0-92.8) | 50.0  (24.7-75.4) | 97.7  (87.7-99.9) |
| DAS-OST (local cut-off for CDAI, <2.71) | 100.0  (66.4-100.0) | 80.0  (66.3-90.0) | 83.1  (71.0-91.6) | 47.4  (24.5-71.1) | 100.0  (91.2-100.0) |
| **Bolean remission as a reference** | | | | | |
| Total HS score (cut-off from literature) | 50.0 (18.7-81.3) | 74.5 (60.4-85.7) | 70.5 (57.4-81.5) | 27.8 (9.7-53.5) | 88.4 (74.9-96.1) |
| Total HS score (local cut-off for boolean remission, <11.0) | 90.0  (55.5-99.8) | 58.8  (44.2-72.4) | 63.9  (50.6-75.8) | 30.0  (14.7-49.4) | 96.8  (83.3-99.9) |
| DAS-OST (cut-off from literature) | 80.0  (44.4-97.5) | 83.7  (70.3-92.7) | 83.1  (71.0-91.6) | 50.0  (24.7-75.4) | 95.4  (84.2-99.4) |
| DAS-OST (local cut-off for boolean remission, <2.73) | 100.0  (69.2-100.0) | 79.6  (65.7-89.8) | 83.1  (71.0-91.6) | 50.0  (27.2-72.8) | 100.0  (91.0-100.0) |

**Supplementary Table 2. Association (regression models with beta coefficients, standard error and p-values) at the patient level, between OST results (Total HS score, DAS-OST and DAS-OST without PtGA) and disease characteristics (multivariate analysis).**

|  | Total HS score | | DAS-OST | | DAS-OST (without PtGA) | |
| --- | --- | --- | --- | --- | --- | --- |
| Variable | Βeta coefficient (SE) | P-value | Βeta coefficient (SE) | P-value | Βeta coefficient (SE) | P-value |
| Number of swollen joints | **0.13 (0.013)** | **<.0001** | **0.44 (0.097)** | **<.0001** | *-0.15 (0.11)* | *0.18* |
| Number of tender joints | **0.071 (0.011)** | **<.0001** | **0.42 (0.066)** | **<.0001** | *-0.10 (0.075)* | *0.17* |
| Health Quality Questionnaire | **0.11 (0.048)** | **0.027** | **1.15 (0.19)** | **<.0001** | *0.079 (0.32)* | *0.81* |
| Patient global assessment - VAS (mm) | *1.00 (0.77)* | *0.20* | **19.9 (2.48)** | **<.0001** | *-2.660 (4.85)* | *0.59* |
| Physician global assessment - VAS (mm) | **2.51 (0.61)** | **0.0001** | **7.88 (2.88)** | **0.0086** | *0.42 (4.02)* | *0.92* |
| DAS28-CRP | **0.12 (0.035)** | **0.0009** | **0.78 (0.14)** | **<.0001** | 0.22 (0.23) | 0.34 |
| DAS28-CRP remission (vs non-remission) | **-0.30 (0.13)** | **0.024** | **-1.93 (0.58)** | **0.0010** | -0.74 (0.51) | 0.14 |
| DAS28-ESR | *0.073 (0.042)* | *0.084* | **1.01 (0.11)** | **<.0001** | **0.74 (0.21)** | **0.0011** |
| DAS28-ESR remission (vs non-remission) | *-0.16 (0.11)* | *0.15* | **-2.39 (0.79)** | **0.0023** | **-2.45 (0.82)** | **0.0028** |
| Boolean remission (vs non-remission) | **-0.53 (0.24)** | **0.030** | **-3.75 (1.64)** | **0.022** | *-0.83 (0.62)* | *0.18* |
| CDAI | **0.080 (0.024)** | **0.0018** | **0.56 (0.10)** | **<.0001** | *0.049 (0.16)* | *0.77* |
| CDAI remission (vs non-remission) | *-0.35 (0.21)* | *0.094* | *-32.3 (22.8)* | *0.16* | *-0.88 (0.66)* | *0.18* |
| SDAI | **0.079 (0.024)** | **0.0015** | **0.56 (0.095)** | **<.0001** | *0.11 (0.16)* | *0.51* |
| SDAI remission (vs non-remission) | **-0.53 (0.24)** | **0.030** | **-3.75 (1.64)** | **0.022** | *-0.83 (0.62)* | *0.18* |
